# Incidental haptic sensations influence judgment of crimes

**DOI:** 10.1038/s41598-018-23586-x

**Published:** 2018-04-16

**Authors:** Michael Schaefer, Lillia Cherkasskiy, Claudia Denke, Claudia Spies, Hyunjin Song, Sean Malahy, Andreas Heinz, Andreas Ströhle, John A. Bargh

**Affiliations:** 1 Medical School Berlin, Calandrellistr. 1, Berlin, Germany; 2 Yale University, New Haven, CT USA; 3 Department of Anesthesiology and Intensive Care Medicine, Charité – Universitätsmedizin Berlin, Berlin, Germany; 4 Arizona Christian University, Phoenix, AZ USA; 5 Stanford University, Stanford, CA USA; 6 Department of Psychiatry and Psychotherapy, Charité – Universitätsmedizin Berlin, Berlin, Germany

## Abstract

Extralegal factors may influence judicial outcomes. Here we investigated the experience of incidental haptic sensations on the harshness of punishment recommendations. Based on recent theories of embodiment, which claim that cognitive representations are structured by metaphorical mappings from sensory experience, we hypothesized that tactile priming with hard objects would cause subjects to recommend harsher sentences (to be ‘hard on crime’). Furthermore, the theory of embodiment predicts that this effect should be based on sensorimotor brain activation during the judging process. In order to test this we presented participants with scenarios that described various crimes while scanning their brain activity with fMRI. Participants were then asked to rate how severely they would sentence the delinquents. Before the scenarios, the participants were primed by touching either a hard or a soft object. Results revealed tha t hard priming led participants to recommend harder punishments. These results were accompanied by activation of somatosensory brain areas during the judging phase. This outcome is in line with simulation assumptions of the embodiment theory and proposes a central role of the sensorimotor cortices for embodied metaphors. Thus, incidental tactile experiences can influence our abstract cognitions and even how hard we are on criminals.

## Introduction

When people are awaiting sentencing after being convicted of committing a crime, we expect that the judges will be fair to them and not be influenced by any factors beyond those of the crime itself. Regardless if one is facing a judge, a juror, a committee, or his or her boss, we assume that no factors external to the misdeeds themselves should influence this interaction. However, we all know that this is often not the case. Several studies report that “extralegal” factors may have an effect on judicial outcomes^1,2^. For example, physical attractiveness of defendants has been shown to be advantageous^3–6^, as is an innocent ‘baby face’^7^. Here we suggest that another factor may influence judicial decision-making. We propose that incidental haptic sensations may have an impact on subsequent judgments.

The supporting theory for this hypothesis is the theory of embodied cognitions, which claims that cognitive representations are structured by metaphorical mappings from sensory experience. In this theory, metaphors are not mere figures of speech, but may actively influence our thoughts and behaviors in an unconscious and often deep way^8^. Numerous studies have found support for this theory. For example, it has been demonstrated that experiencing physical warmth as through holding a cup of hot (versus iced) coffee makes individuals more likely to judge someone as having a “warm” personality^9^. Additionally, holding something warm activates the same small region of the insula as when the person sends or receives a text from a family member or friend (i.e., social warmth)^10^. Moreover, recent studies have shown that the activation of concepts such as ‘hardness’ through actual physical experiences may guide analogue psychological concepts. These abstract concepts are based on idioms such as “hard-hearted” or “hard day” and link ‘hardness’ with metaphorical meanings of resistance to influence. For example, Ackerman *et al*. asked participants to imagine shopping for a new car, making an offer to the dealer, being rejected, and then being asked to make a second offer. Half of the participants were sitting in a hard wooden chair, the other half in a soft cushioned chair. Those participants sitting in a hard chair made a smaller adjustment to their offer. Thus, they ‘took a harder line’ in this negotiation^11^.

Where do these physical-to-psychological priming effects come from? It has been suggested that early experiences with the physical world structure or ‘scaffold’ our later understanding or representation of more abstract concepts^8,12,13^. Hence, abstract concepts such as ‘hardness’ may be based on, and associatively connected to, representations of sensory and motor experiences^14–16^. The conceptual metaphor theory holds that mental processes involve simulations of body-related perceptions and actions. Hence, the retrieval of conceptual meaning involves a partial re-enactment of sensory and motor experiences. Conceptual metaphor theory would thus predict that any effects of “hard” priming on psychological concepts should involve sensorimotor brain regions being active during the judgment phase and influencing those judgments accordingly.

The current study had two aims. First, we examined if the activation of the psychological concept of ‘hardness’ may also affect judgment decisions. Thus, we tested whether incidental “hard” priming led people to be “hard” on crimes. This hypothesis is based on recent findings that negotiators seated on hard chairs do not compromise as much as do those seated on soft chairs^11^. Second, we evaluated whether the neural underpinnings of this effect are found in the sensorimotor cortices, which would strengthen the hypothesis of the physical-to-psychological link hypothesized by the conceptual metaphor theory.

The first study report results of a behavioral pre-study that manipulated hard and soft experiences (sitting on a hard vs. sitting on a soft chair) while participants were asked to judge crime scenarios. The second study aimed to explore the neural correlates for this effect by using an fMRI approach.

## Study 1

We predicted across two experiments, using 6 different hypothetical criminal scenarios and diverse response formats, that sitting in a hard chair as compared to a soft chair would cause participants to punish hypothetical criminals more harshly because the hard chair should automatically and implicitly (without the participants’ awareness) activate the concept of harshness while the soft chair should activate the concept of leniency.

We additionally investigated the potential mediational role of changes in emotions or political attitudes produced by the chair hardness manipulation on sentencing behavior. It is possible that any effect of hard (versus soft) chairs on punishment severity might operate by temporarily influencing the participants’ political and social views to be more conservative^17–19^. It is also possible that the influence of chair hardness on sentencing may operate by making participants in the hard (versus soft) chair condition experience more negative emotions because the hard chair may be uncomfortable or unpleasant to sit in. We wanted to investigate this possibility because negative emotional states have been previously linked to harsh (versus lenient) judgments, including anger^20^ and sadness^21^.

### Experiment 1: Sitting in hard chairs makes people hard on thieves and murderers

In Experiment 1, 41 students (mean age = 19 years, 25 females) were randomly assigned to complete a paper and pencil questionnaire at a desk in the laboratory while seated in either a hard wooden chair or a soft cushioned chair. These chairs were identical to those used by Ackerman *et al*.^11^. The questionnaire contained four short hypothetical crime scenarios (adapted from^22^) which describe 1) a youth who stole a T.V., 2) a bank teller who falsified accounts and stole money, 3) a man who stabbed his unfaithful wife, and 4) a repeat offender who robbed a bank with accomplices and killed a guard. For each scenario, participants were asked to give their judgment as to how many months (if any) the offender should spend in jail and additionally how much money (if any) he or she should be fined for the crime. Next, participants completed the Positive and Negative Affective Schedule (PANAS)^23^, which assesses the extent to which they currently feel each of 20 emotions, on scales from 1(not at all) to 5(extremely); following which they were debriefed and thanked for participating.

To prepare our data for analysis, we first converted into z-scores participant judgments of how many months the offender should spend in jail and how much money he or she should be fined for the crime. Because combined, these two judgments constitute the full recommended punishment for each described crime, we added together the two z-scores for jail time and fine amount for each scenario to create one composite punishment score from each participant for each scenario. We added the z-scores instead of averaging them because conceptually, the jail time and fine variables are additive components of the overall assigned punishment for each hypothetical criminal rather than redundant measures of the same conceptual variable.

**Figure 1:**
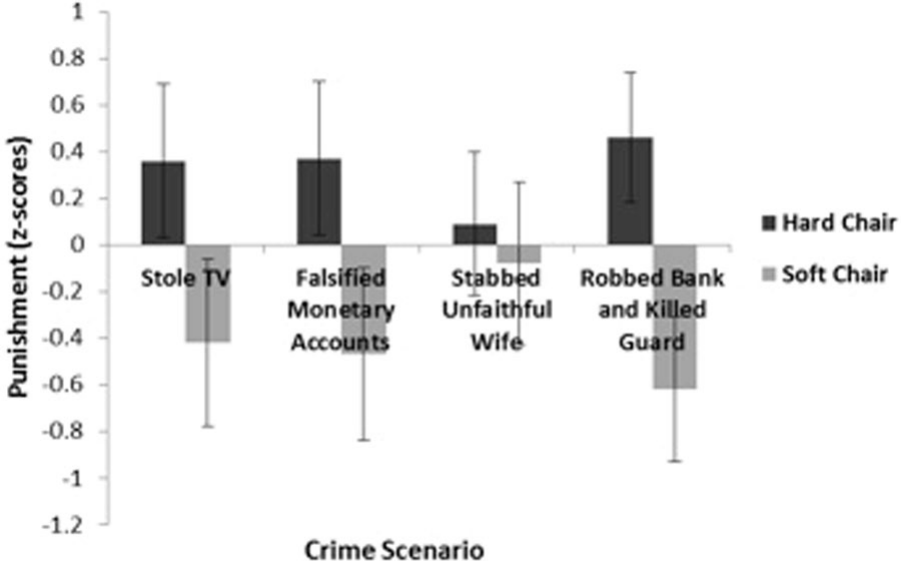
Recommended punishment for scenarios involving thieves and murderers is shown as a function of chair condition (study 1, experiment 1). Error bars represent ± 1 SEM.

A 4 (criminal scenarios 1–4) × 2 (hard vs. soft chair) repeated measures analysis of variance (ANOVA) on punishment harshness revealed a main effect of the chair hardness manipulation. As predicted, compared with participants in the soft chair condition, participants in the hard chair condition assigned harsher composite punishments across the 4 scenarios (F(1, 39) = 5.03, p < 0.05, see Fig. 1) (the pattern of results is the same when recommended jail time and fine are each analyzed separately using a repeated measures ANOVA).

Next, we assessed whether changes in the valence of experienced emotions mediated the influence of chair hardness on punishment harshness. Following standard scoring procedure, the PANAS score was obtained by subtracting the total negative emotion score from the total positive emotion score to calculate a net positive emotional valence score. Chair hardness had a marginal effect on net positive emotional valence scores, t(36) = 1.91, p = 0.07, but in the opposite of the predicted direction, with participants seated in the hard chair experiencing more positive emotions (M = 14.52, SD = 5.82) than participants seated in the soft chair (M = 10.29, SD = 7.86). There was no influence of emotional valence on composite punishments for any of the 4 scenarios (largest r = 0.25, p > 0.14), so a test of mediation by emotional valence of the effect of chair hardness on punishment was not justified.

### Experiment 2: Sitting in hard chairs makes people hard on cheaters

We had three goals for Experiment 2: 1) replicate the main effect from Experiment 1 using criminal scenarios involving less severe crimes, 2) replicate the unexpectedly positive (albeit marginal) influence of chair hardness on emotions from Experiment 1 using a different measure of emotional valence, and 3) investigate an additional potential mediator of the relationship between chair hardness and punishment harshness: change in political attitudes.

In Experiment 2, 44 students and community members (mean age = 22 years, 33 females, 2 unspecified) were randomly assigned to sit in the hard or soft chair as in Experiment 1 while completing a paper-and-pencil questionnaire on a clipboard. Participants were recruited and completed the short (3 minute) study at a busy indoor intersection on a college campus. The questionnaire contained two original hypothetical scenarios about two college students who cheated; one who had failed to cite heavily referenced sources in a final course paper that earned 91/100 points, and another who had copied another student’s answer on a midterm exam on which she earned 94/100 points. For each scenario, participants were asked to give their judgment as to how many points should be deducted as a punishment for cheating on the assignment. Next, participants answered “How positive do you feel right now?” on a scale from 1 (Not at all) to 7 (Extremely) and “How negative do you feel right now?” using the same scale. Finally, participants were administered the measure of liberal versus conservative attitudes, in which they indicated how they would describe their political beliefs 1) overall, 2) socially, and 3) economically, using the following scale for each item: 1(Very liberal) to 9 (Very conservative).

To prepare our data for analysis, we first checked whether the points deducted punishment variable was normally distributed for each scenario using a Kolmogorov-Smirnov test for normality^24^ which showed that the distributions for both scenarios were non-normal (smallest d = 0.24, p < 0.001). Accordingly, we converted points deducted for both scenarios into z-scores to correct for this severe level of non-normality. The two items measuring emotional valence were combined using a procedure analogous to PANAS scoring in which the negativity item was subtracted from the positivity item to create an index of net positive valence. Finally, the three items measuring overall, social, and economic political attitudes were averaged to form a composite index of political attitude (α = 0.80). A Kolmogorov-Smirnov test for normality revealed that the distribution for the composite political attitude variable was non-normal (d = 0.14, p < 0.05) so we applied the natural log transformation to it (new d = 0.11, p > 0.19.)

**Figure 2:**
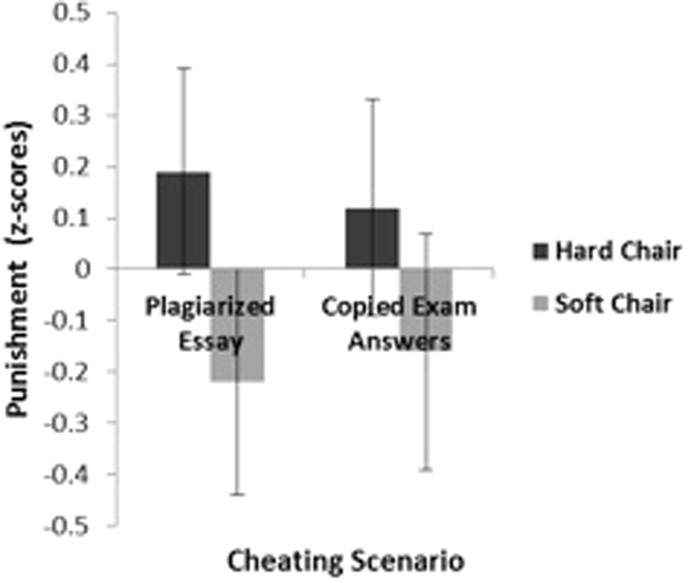
Recommended punishment for scenarios involving academic cheating is shown as a function of chair condition (study 1, experiment 2).

As predicted, a 2 (cheating scenarios 1–2) × 2 (hard vs. soft chair) repeated measures analysis of variance (ANOVA) on punishment harshness (operationalized as amount of points deducted) revealed a main effect of the chair hardness manipulation. Compared with participants in the soft chair condition, participants in the hard chair condition recommended deducting more points across both scenarios (F(1,42) = 4.74, p < 0.05, see Fig. 2). Next, we tested whether sitting in a hard chair would, as in Experiment 1, produce the experience of more positive emotions compared to sitting in a soft chair. In Experiment 2, however, chair hardness did not influence emotional valence, t(45) = 0.45, p > 0.60.

Chair hardness did have the predicted significant effect on political attitudes, t(44) = 2.15, p < 0.05, with participants in the hard chair reporting more conservative attitudes (M = 4.10, SD = 1.53) as compared to participants in the soft chair (M = 3.28, SD = 1.55). There was no influence of political attitudes on points deducted for either of the 2 scenarios (largest r = 0.07, p > 0.64), so a test of mediation by political attitudes of the effect of chair hardness on punishment was not justified.

## Study 2

Study 2 aimed to examine the neural underpinnings of this ‘hard on crime’ effect. Since passive tactile stimulation using different chairs (or underlying mattresses) is difficult to manipulate inside an MRI, we changed the paradigm into a within-subjects design with active tactile stimulation. Participants were asked to read scenarios describing a series of different crimes while we scanned their brain activity with fMRI. After each scenario, participants had to rate what sentence they would give the delinquent. Prior to each scenario, the participants were primed with a hard or a soft object. We examined whether hard priming would cause the participants to be “hard” on crime and if this effect would be based on activity in the sensorimotor cortices.

## Materials and Methods

### Participants

Seventeen people (12 females) with a mean age of 24 years (standard deviation +−3.58, range 18–31) took part in the study. All participants were right-handed native German volunteers with no neurological or psychiatric history. The participants gave written informed consent to the study, which adhered to the Declaration of Helsinki and was approved by the ethical committee of the University of Magdeburg, Germany (ethical committee: 133/12, clinical trials: NCT02517060).

### Procedure

Participants were told that they would perform two separate experiments in the session: an experiment in order to examine neural correlates of touch experiences and an experiment to investigate neural correlates of judgments processes (cover story).

The study design included one factor, tactile priming, which was hard, soft, or omitted (no tactile stimulation). Priming was done by using soft (foam material) or hard (wood) objects (comparable weight, seize and shape). At the beginning of the priming phase an experimenter placed the hard or soft object to the participant’s hand in a way that he or she was able to feel the object between his or her thumb and other fingers. The participant did not hold the object in his or her own hand. Hence, the participant was not able to see the objects, freely explore the object, feel the shape or the weight of the object, or swipe on the objects to assess the surfaces. He or she was only allowed (and clearly instructed before) to repetitively press and feel the hardness or softness of the objects by using thumb and residual fingers, while this object was hold by the experimenter. Prior to the beginning of the experiment we made the participant familiar with the task.

While horizontally reclined in the scanner the participants received one of the priming stimuli (a hard, a soft, or no object). Participants were allowed to feel the hardness of those stimuli by repetitively touching them for about 15 seconds. After this priming, participants were prompted with a screen describing a crime scenario. These scenarios covered crimes such as burglaries, criminal assaults, murderers, as well as cheating and drug offences. All scenarios were ambivalently valenced in order to prevent straightforward judgments. Thus, the scenarios included both positive (mitigating) and negative components. For example, participants read the following scenario: “A twenty-year-old man drove his friends to a night club. Because he was intoxicated and the atmosphere in the car was distracting, he ran a red light, thereby causing a serious traffic accident. Later on, the young driver apologized for his actions when meeting the injured persons”. The presentation of the scenario lasted for 16 seconds. The subsequent screen asked the participants to give their judgment as to how seriously the protagonist should be punished (“How seriously should the young man be punished? More seriously: right buttons. Less seriously: left buttons”). Participants used two keys with four buttons for each hand (8-point Likert-scale ranging from 1 to 8) to judge the crimes. Before the experiment, they were told that they could weight their responses from moderate (inner buttons) to extreme (outer buttons). Use of right and left buttons was randomized over the scenarios. Participants had 14 seconds to give their judgment (earlier responding did not automatically start the next trial). Then there was a break of 12 seconds until the next trial started. We used brain activity in a time window around the button press in order to assess comparable parts of the judgment process for all participants. Hence, condition-related activity was measured using an individual “floating” time window of eight MR images (four images before, one during, and three after the button press, resulting in 16 seconds) covering the time of the point of response^25^.

A total of 60 scenarios was shown to each participant. The order of presentation of the scenarios as well as the kind of priming for the scenarios (hard, soft, or no object) was randomized between and within the subjects. Prior to the beginning of the experiment we made the participants familiar with the task.

Visual images were back-projected to a screen at the end of the scanner bed close to the subject’s feet. Subjects viewed the scenarios through a mirror mounted on the birdcage of the receiving coil. Foam cushions were placed tightly around the sides of the subject’s head to minimize head motion. The experiment consisted out of four runs, each lasting for about 14 minutes. Each run included all conditions. Participants were allowed to take short breaks between the runs.

After scanning, the participants were probed for suspicions concerning the experimental hypotheses (“What do you think was the purpose of this study?”, “Do you have any ideas about the hypotheses of this study?”). Finally, they were debriefed and thanked for participating.

### FMRI Data Acquisition and Analysis

Functional scans were acquired by using a 3T scanner (Siemens MAGNETOM Trio, Germany) (gradient echo T2-weighted echo-planar images; TR = 2 sec, TE = 30 ms, flip angle = 80 degrees, FOV = 192 mm). For each subject, data were acquired in four runs. In each session, 416 volumes were acquired. Functional volumes consisted of 32 slices. Each volume comprised 3.5 mm slices (no gap, in plane voxel size 3.5 × 3.5 mm). For anatomical reference a high-resolution T1-weighted structural image was collected for anatomical reference (MPRAGE, TR = 1900 ms, TE = 2.5 ms).

FMRI data was preprocessed and analyzed using the Statistical Parametric Mapping Software (SPM, Wellcome Department of Imaging Neuroscience, University College London, London, UK). For each subject, the fMRI scans were realigned to correct for inter-scan movement, using sinc interpolation and subsequently normalized into a standard anatomical space (MNI, Montreal Neurological Institute template), resulting in isotropic 3 mm voxels. The scans were then smoothed with a Gaussian kernel of 6 mm full-width half maximum.

We then computed statistical parametric maps by using multiple regressions with the hemodynamic response function modeled in SPM. Data analyses were performed at two levels. First, we examined data on the individual subject level by using a fixed effects model. Second, the resulting parameter estimates for each regressor at each voxel were entered into a second-level analysis with the random effects model.

To examine brain responses while participants explored the stimuli, we computed statistical contrasts (t-tests) for hard stimuli relative to no stimulation (fixation cross) and soft stimuli relative to no stimulation.

In order to investigate brain activity during the judgment process, we examined the time window while the participants gave their recommendations for punishments. We computed an ANOVA for repeated measurements for the priming factor (soft, hard, none). Subsequently, statistical contrasts (t-tests) were performed to examine cortical activation during the judgment depending on the different priming conditions. Behavioral responses (judgment scores) were used to test for possible correlations (Pearson) with the parameter estimates for voxels in the sensorimotor regions of interest (maximum peaks in bilateral primary somatosensory cortex (SI)).

We report regions that survived correction for multiple comparisons over the whole brain (at p < 0.05, family-wise (FWE) correction). Furthermore, in order to test our hypothesis that the “hard on crime” effect is grounded in sensorimotor activations, we report regions of interest that survived a small volume correction (SVC) of p < 0.05 (FWE corrected at the peak level) for which we had an a priori hypothesis. Thus, an SVC was applied to activations within a sphere of 15 mm radius in the left and right SI. The coordinates of these SVCs resulted from the analysis of contrast of hard and soft stimulation relative to no stimulation during the exploration phase (peak activations in SI).

Anatomical interpretation of the functional imaging results was performed using the SPM anatomy tool-box^26^.

## Results

### Behavioral results

None of the participants reported any suspicions with respect to our experimental hypotheses. In particular, none of the participants guessed the relation between the tactile and the judgment tasks. All of them believed to have participated in two separate experiments.

Two participants were excluded prior to data analysis; one due to technical reasons (technical problem of behavioral data collection) and the other due to lack of behavioral responses after beginning (response device did not collect behavioral responses after the first minutes of the experiment).

**Figure 3:**
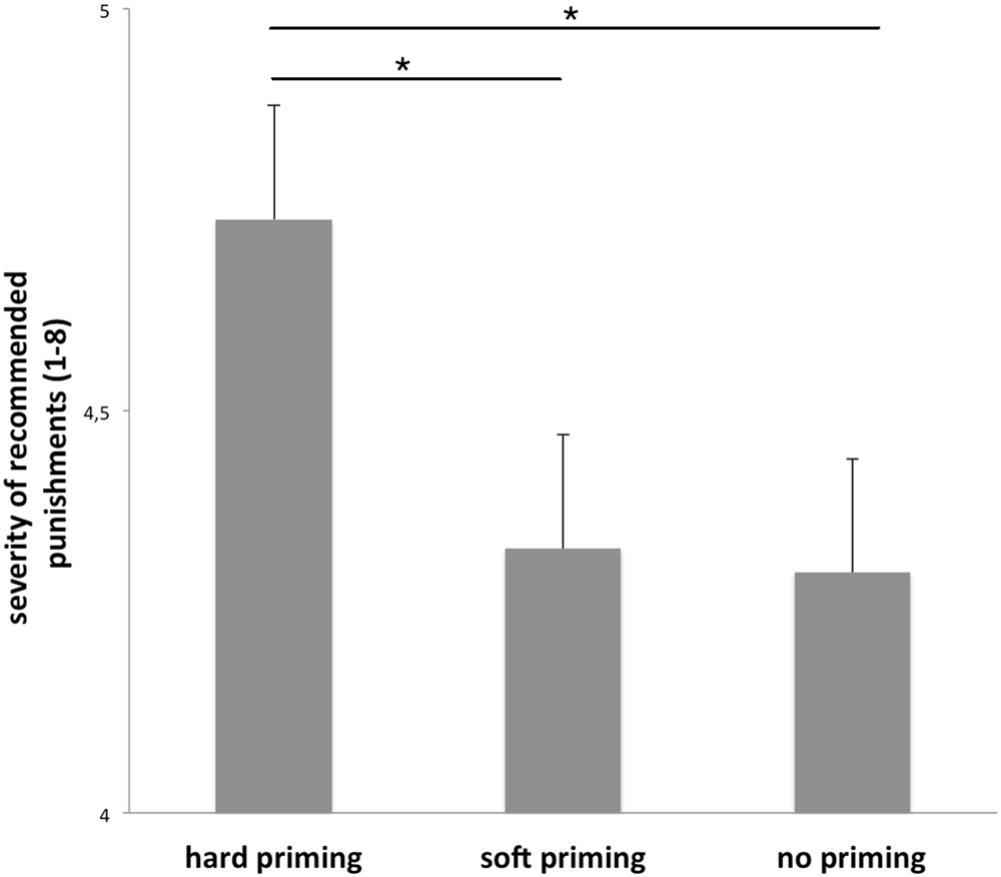
Participants’ mean recommendation for sentences (+standard errors) after soft, hard, or no priming (scale from 1 to 8, with 8 for very hard sentences). Hard priming resulted in significantly harder punishment recommendations.

Analysis of the behavioral results (ANOVA with factor priming: hard, soft, no) revealed a significant effect (F (2,28) = 3.73, p = 0.03). Post hoc t-tests showed that judgments of criminal severity were more serious after hard priming (4.77 ± 0.94, mean and standard deviation) compared with soft priming (4.36 ± 0.63; t(14) = 2.11, p = 0.02) and compared with no priming (4.39 ± 0.56; t(14) = 2.16, p = 0.02). Judgments after soft priming were not different compared with no priming (t(14) = 0.21, p > 0.10) (see Fig. 3). Analysis of the reaction times revealed no significant effects.

### FMRI results: Brain responses while exploring the tactile stimuli

**Figure 4:**
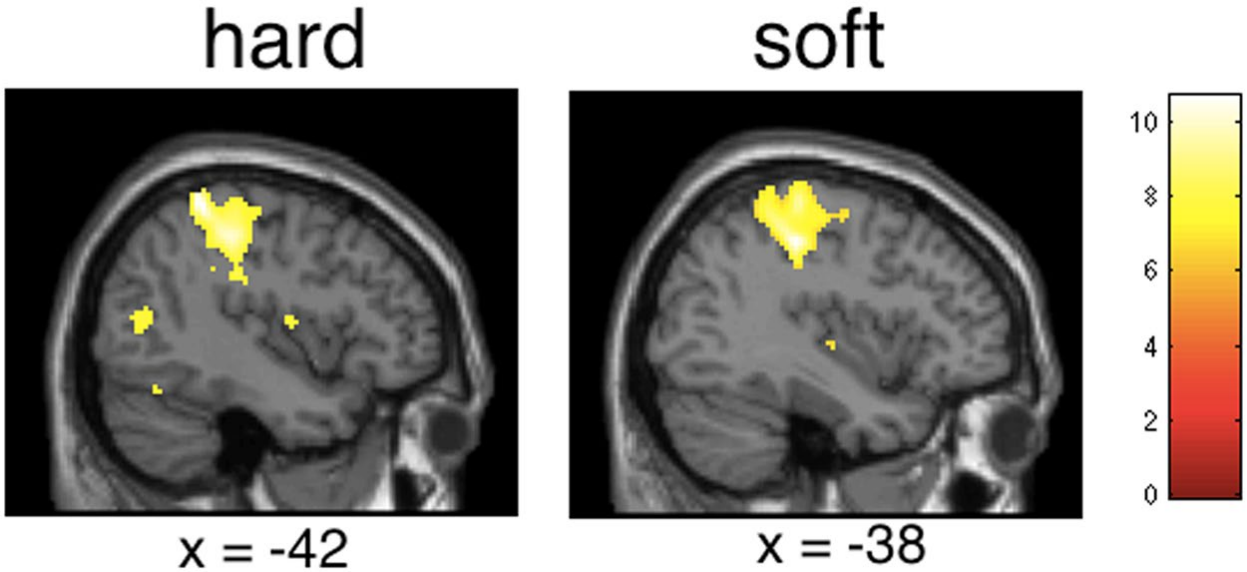
Statistical maps showing brain activation while participants explored the hard and soft primes, respectively (relative to rest, FWE corrected). Areas of significant fMRI signal change are shown as color overlays on the T1-MNI reference brain.

Brain responses when actively exploring the priming stimuli (hard relative to rest and soft relative to rest) showed activation of sensorimotor brain areas (primary and secondary somatosensory cortices, primary motor cortex, premotor cortex) and other areas, p < 0.05, FWE corrected), as expected (see Fig. 4).

Comparing brain activations during hard relative to soft stimulation at the whole brain level revealed activation in bilateral sensorimotor cortex and other brain areas (p < 0.05, FWE corrected at the cluster level) and other brain areas (uncorrected). Brain responses while handling the soft object relative to handling the hard object failed to show any significant activation.

### FMRI results: Brain responses while assessing crime scenarios

**Figure 5:**
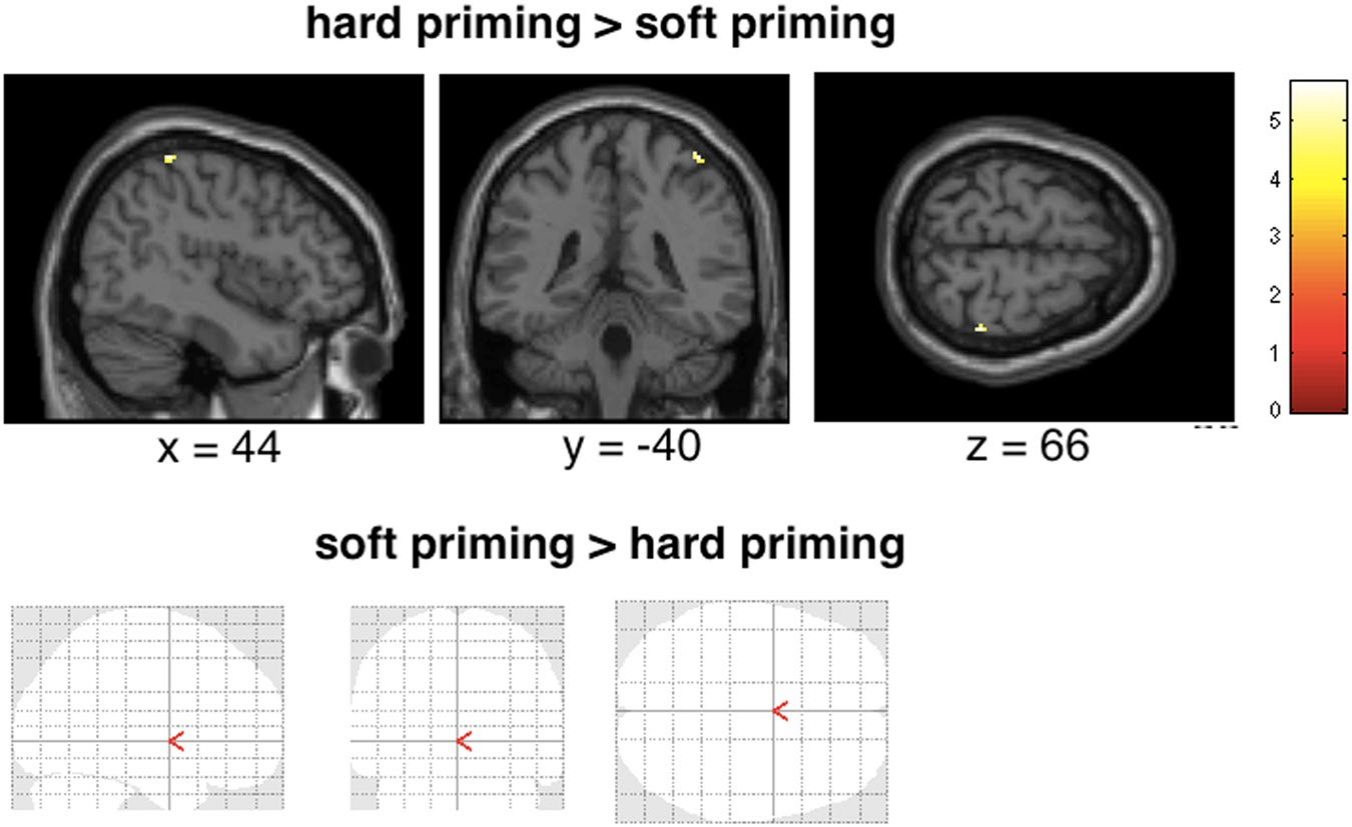
Statistical maps showing brain activation while participants recommended sentences (whole brain analysis). Hard relative to soft priming revealed brain activation in somatosensory cortex (no other brain areas activated, see Table 1). Soft relative to hard priming revealed no significant activation (see Table 1).

We then examined brain areas of the participants that behaviorally already showed the “hard-on-crime” effect in order to investigate the neural underpinnings of this effect. Thus, we examined brain responses while the participants were giving their recommendations of sentences after having read the scenarios. Analysis of brain activations revealed engagement of sensorimotor brain regions (SI, SII, BA6) and inferior frontal gyrus (ANOVA main effect, factor priming; hard, soft, no). Post hoc t-tests demonstrated that brain responses while participants recommended sentences after having explored the hard object compared with being primed with the soft object involved activation in SI (at p < 0.05, FWE corrected, see Table 1 and Fig. 5). No other brain areas were activated (even at an uncorrected threshold, see Table 1). Brain responses during judging when being primed with the hard object compared with no priming at all showed again activation in SI, but only at an uncorrected threshold (see Table 1). Furthermore, premotor cortices, secondary somatosensory cortices, and inferior frontal cortex were engaged (at an uncorrected threshold, see Table 1). The contrasts of brain responses during judging for soft priming relative to hard priming and for soft priming relative to no priming failed to show any significant voxels (at p < 0.05, FWE corrected).

**Table 1:** Results of random effects analysis for brain responses when recommending sentences depending on different priming conditions (p < 0.05, FWE corrected, L = left hemisphere, R = right hemisphere; in brackets: uncorrected results). See text for further details.

Contrast	Brain region	Peak MNI location (x, y, z)	Peak z-value	Number of voxels
hard > soft priming	R SI (R SI) (L SI)	44 −40 66 60 −32 54 −58 −38 52	3.80 2.94 2.88	32 12 16
soft > hard priming	—	—	—	—
hard > no priming	(L SI) (L BA6) (R BA6) (R SII/Insula) (L SII/Insula) (R inf. frontal gyrus/BA44) (L temporal gyrus) (L middle frontal gyrus/BA45)	−24 −26 50 −32 −4 38 22 −16 58 40 −12 2 −28 8 16 62 12 6 −56 −10 2 −24 −28 56	3.50 3.70 3.39 3.46 3.28 3.35 3.34 3.15	19 8 12 31 7 6 6 7
no > hard priming	—	—	—	—
soft > no priming	(L inf. frontal gyrus/BA44)	−40 8 14	3.33	10
no > soft priming	—	—	—	—

**Figure 6:**
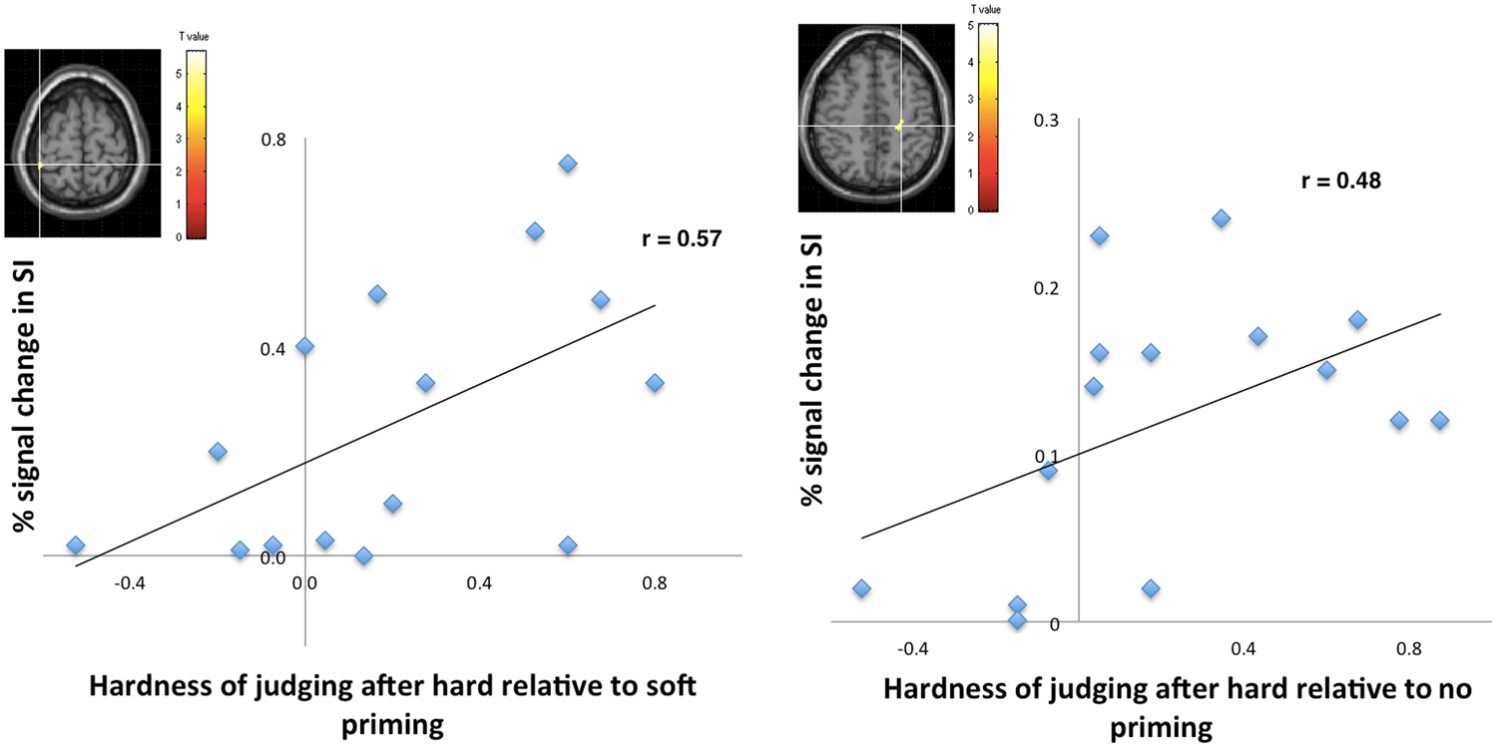
Brain activation in somatosensory cortex after hard priming could significantly predict the strengths of the hard on crime effect (peak activations in SI, x = 44, y = −40, z = 66 and –x = −24, −y = −26, z = 50). See text for further details.

In order to further test our hypotheses we then computed correlations of the strength of the neural activation in somatosensory cortices and the behavioral responses of all participants. Thus, we calculated correlations between the strength of the hard on crime effect (behavioral data: judgment scores after hard priming minus judgment scores after soft priming) with signal changes in somatosensory peak areas for the contrast rating after hard priming relative to rating after soft priming. Results revealed a significant correlation of the strength of the “hard on crime” effect with signal change in SI (r = 0.57, p < 0.05, Pearson, see Fig. 6). For the comparison between judging after hard priming relative to judging after no priming, signal changes in SI were also significant positively linked to the strength of the hard on crime effect (r = 0.48, p < 0.05, Pearson). Thus, the more the participants were affected by the ‘hard on crime’ effect, the more their somatosensory cortices were engaged in the later judgment phase. Thereby, the data suggests that the observed SI activation is linked to the ‘hard on crime’ effect. Other areas did not show any significant correlations with the ‘hard on crime’ effect.

In order to test if even punishment judgments without any priming may correlate with sensorimotor activations, we contrasted brain responses while participants were giving their recommendations of sentences without any priming with activation during baseline. Results revealed neural activations in occipital and posterior parietal lobe, sensorimotor brain regions and frontal lobe. However, given that we here compared brain activation during reading, judging, and pressing a button with a baseline condition, we cannot draw strong conclusions out of this comparison. A correlation between neural responses in sensorimotor cortex and behavioral responses (punishment ratings) revealed no significant correlation.

## Discussion

People are often judging the behaviors of others in a legal capacity, for example as a judge or member of a jury. While we assume that these processes are fair and consider only legal aspects of the relevant situations, we know that in real life there are numerous situations in which so called extralegal factors are relevant. Here we aimed to experimentally investigate the influence of incidentally and briefly explored hard and soft objects on subsequent judgments of the severity of crimes. Results of a behavioral study demonstrated that sitting in hard chairs make people harder on crime. These results are confirmed by an fMRI study, showing that experiencing “hard” vs. “soft” objects before judging the severity of crimes leads to “harsher” judgments (compared to soft priming or no priming). Thus, “hard” priming makes us harder on crime. In contrast, “soft” (vs. no) priming did not induce any significant effects. FMRI results revealed that this “hard on crime” effect was based on the primary somatosensory cortex.

### The psychological concept of hardness

Our results are in line with other studies, which found evidence that haptic sensations can prime later assessments, attitudes, or behavior. For example, Ackerman *et al*. (2010) reported that negotiators sitting in hard chairs did not compromise as much as did those sitting in soft chairs. Similarly, they also found that participants primed with handling a hard object subsequently judged an employee to be more rigid or strict (in contrast to priming with a soft piece of blanket). Thus, both active as well as passive haptic sensations of hardness affected later social assessment tasks. These physical-to-psychological priming effects are in line with the “hardness” metaphor, which associates physical “hardness” with terms or metaphors such as “hard-hearted”, “hard times”, or “hard on crime”. Notably, Ackerman *et al*. found an effect on rigidity of the employee, but no general positive (or negative) effects on the ratings. This is in line with the conceptual metaphor theory that claims that metaphors are more than mere abstract linguistic figures. According to Lakoff and Johnson metaphors influence our thoughts, feelings, intentions and behavior in an unconscious but often deep way^8^. Our results support this theory and show that priming the participants with an active experience of a hard object made them harder on crime relative to soft priming or no priming at all. This is demonstrated by behavioral results, showing that the experience of hard objects (sitting in a hard chair) made participants harder on crime, but did not make the participant generally feel more negatively. Hence, “hardness” had an effect on judgments of criminal severity, thus documenting an extralegal factor in judgment processes.

Interestingly, we did not find any effects of soft priming vs. no priming on later judgment processes, either for the behavioral data (study 2) nor for the imaging data. One could speculate that softness may not be similarly embodied as hardness. Another possibility is that the negative pole of dimensions has stronger and more easily detectable influence than the positive pole, assuming soft is more positive than hard. For example, in their demonstration of the similarity of physical and social (betrayal in economic game) temperature effects, Kang *et al*. found greater activation of the bilateral insular-opercular cortex caused by cold sensations compared to neutral temperature but no difference in activation levels between the warm and neutral conditions^27^. This is in line with the general finding that ‘bad is stronger than good’ in their influences on social judgment^28^. It also might be that in our study the soft stimuli were not sufficiently soft to elicit an effect. Further studies are needed in order to replicate this ‘hard-but-not-soft’ finding.

### Neural basis of the ‘hard on crime’ effect

Our study also revealed the neural underpinnings of “hard on crime” effect. The theory of embodied cognition makes clear assumptions of an interaction between body and mind^8,15^. Neuroimaging tools provide excellent access in order to test these assumptions. Several studies support the embodiment theory by proving the assumption that motor and in particular somatosensory cortices are crucial neural correlates of embodied cognitions. For example, it has been shown that comprehending textural metaphors activated the somatosensory cortex^29^. Moreover, several studies reported sensorimotor activation during language comprehension in the action domain, thereby suggesting sensorimotor circuits as a cortical basis for language^30^. Furthermore, it has been demonstrated that the moral-purity metaphor^31^ is associated with activations in sensorimotor brain regions^25,32^. In addition, the roughness metaphor (rough stimuli prime subsequent interactions to become an argument rather than a discussion) activated particularly sensorimotor brain areas^33^. Our results are in line with these reports. The “hard on crime” effect was predicted by the activation of the somatosensory cortex: the more the somatosensory cortex was engaged, the stronger the effect. We therefore conclude that these results support the embodiment theory^8,12,14^.

The current experiment focused on punishment judgments depending on different tactile priming stimuli. While we report effects of this priming on punishment judgments, it would be very interesting to test if also other measures were affected by the haptic priming. For example, the priming might had have a general impact on mood or generic positive (or negative) thinking. However, the present study did not include control questions due to time reasons (in order to maximize signal strength in the fMRI). Nevertheless, we think that it is unlikely that the priming might had have a general effect on mood or positive feelings, because the sensorimotor cortex, which we show to be associated with the “hard on crime” effect, is not known to be related to feelings such as positive moods. Moreover, results of our pre-study investigated two possible mediators of the influence of hardness on punishment harshness: emotional valence and political attitude. Although we did not find mediation of the effect on punishment by either variable, or consistent results for the influence of hardness on emotional valence, we did find that participants seated in hard chairs consistently reported more conservative (versus liberal) attitudes, compared to participants seated in soft chairs. This finding contributes to a growing body of work suggesting that political attitudes may be more malleable than previously thought (e.g.^34^). This finding is also in line with previous research documenting the influence of tactile primes on social attitudes^11^.

We here argued that the retrieval of conceptual meaning involves a partial re-enactment of sensory and motor experiences. Based on this assumption we aimed to demonstrate that tactile priming would in particular enhance this engagement of sensorimotor brain regions. However, according to our theoretical considerations we also assume that even without priming hard punishment judgments should be associated with activations of sensorimotor brain regions^29,30^. The present study did not find a significant correlation between sensorimotor activation and judgments without any priming. This may be explained by weaker activations in sensorimotor activations when not being primed with hard haptic experiences. Nevertheless, the present experimental design is less suited to examine sensorimotor activation without any tactile priming. Future studies are needed to assess this hypothesis.

### The role of neuroscience in research on embodied cognition

Why is a neuroscientific approach important for the debate of the theory of embodied cognition? What do we gain by this perspective? Neuroscientific evidence provides us with information on the neural underpinnings of behavioral effects. This seems particularly important within the perspective of embodied cognition. For example, in the traditional understanding knowledge is represented abstractly in an amodal or supramodal conceptual network of formal logic symbols^35^. The theory of embodied cognition challenges this view and claims that cognitive representations that constitute our knowledge are grounded in sensory and motor experiences^8,16^. Neuroscientific approaches seem to be an excellent tool to test this hypothesis of a sensorimotor grounding. Thus, the present results demonstrate that even highly abstract thinking (punishment judgments related to crimes) is accompanied by activations of somatosensory brain areas. In contrast, the traditional understanding would have hypothesized that abstract cognitions are based on frontal brain areas, regions not known to be related with basic sensorimotor processing and perception. Furthermore, data from brain imaging may also add a new level of data to theoretical considerations that primarily are based on behavioral data. Thereby, neuroimaging data also allow us to link the embodiment theory to other neuroscientific theories. For example, the present results report predominantly activation in primary somatosensory cortices. These brain areas are also described as being related to mirror neurons and empathic personality traits^36,37^. Those cross-references may help us to further understand how brain and behavior are related. Last, neuroscientific evidence also opens the way to further establish the assumptions of the theory of embodied cognitions. For example, if sensorimotor brain areas are essential for abstract thinking, the temporary blocking of these brain areas (so-called virtual lesions) by using the transcranial magnetic stimulation (TMS) approach should prevent the effects we reported here and also diminish abstract thinking in general. Thus, given that several fMRI approaches stressed the role of sensorimotor brain areas for embodied cognition^25,33^, TMS could provide complementary results.

### Conclusions: Implications

Previous research has shown that hypothetical sentencing scenarios can be generalizable to real-world scenarios^38^. Based on the results of Ackerman *et al*. and the present study, we suggest that incidental haptic experiences such as hardness of objects or even furniture (chairs) may influence judgments rendered in actual courtrooms. Future studies should examine the extent of this effect on punishment severity, for example with respect to the duration of the effect (do the effects of sitting in a hard chair immediately disappear upon standing up?). Furthermore, our study consisted of average lay people, not experienced judges and jurists. It remains to explore whether persons who are trained to judge situations according to certain guidelines are also prone to this “hard on crime” effect. However, there seem to be numerous real world examples documenting that even very experienced judges are not completely immune to extralegal factors (e.g., the field studies in California courtrooms by Konecni and Ebbesen)^39^, which suggests that even highly trained individuals may be susceptible to such effects.
